# What motivates and demotivates emergency response volunteers? A survey-based factor analysis study

**DOI:** 10.1186/s13049-023-01101-0

**Published:** 2023-08-12

**Authors:** Erik Prytz, Petter Norrblom, Sofie Pilemalm, Tobias Andersson Granberg, Carl-Oscar Jonson

**Affiliations:** 1 Department of Computer and Information Science, Linköping University, Linköping, Sweden; 2 Department of Science and Technology, Linköping University, Linköping, Sweden; 3 Center for Disaster Medicine and Traumatology, and Department of Biomedical and Clinical Sciences, Linköping University, Linköping, Sweden; 4 Department of Information Systems, University of Agder, Kristiansand, Norway

**Keywords:** Volunteers, Motivation, Cardiac arrest, Emergency response

## Abstract

**Background:**

Organized volunteer initiatives can reduce response times and improve outcomes in emergencies such as cardiac arrests or fires. Retention of volunteers is important to maintain good coverage and capabilities. The current study explores factors underlying volunteers’ motivation to continue as volunteers.

**Methods:**

Data from 5347 active volunteers were collected through an online survey. An exploratory factor analysis was used to identify underlying factors that were then used in a regression analysis to predict intention to continue as a volunteer. Group differences based on, among others, number of alarms and prior professional experience in emergency response were explored.

**Results:**

The results showed that the factors community, self-image, and competence were the strongest positive predictors for the motivation to continue, whereas alarm fatigue and negative experience were the strongest negative predictors. Volunteers with professional background had higher competence and lower Alarm fatigue. Volunteers from rural areas and small cities had higher community than those in large cities.

**Conclusions:**

Alarm fatigue can make it hard to retain volunteers, which could be addressed using improved dispatch algorithms. Support after dispatch is important to prevent negative experiences. Finally, increased competence, e.g. through education and training, can improve volunteer’s motivation to continue.

**Supplementary Information:**

The online version contains supplementary material available at 10.1186/s13049-023-01101-0.

## Background

In the last 20 years, resources not formally part of the traditional emergency response services, such as semi-professionals, laypeople, and volunteers, have been increasingly used to provide complementary support in emergencies [1]. To make good use of these new actors, many countries have implemented initiatives using volunteers providing aid prior to the arrival of emergency services to decrease response times and improve outcomes [2].

Retention of active volunteers is important to sustain a pool of volunteers who can provide this type of support. Retention is also important for expanding existing initiatives to provide better coverage and enable quicker response times. Two aspects that need to be taken into consideration when it comes to retention are the experiences and motivation of volunteers. Because volunteers do not have obligations to continue volunteering, having a negative experience of the volunteering role or not having the motivation for volunteering fulfilled could lead to high attrition rates. Several studies have been conducted in the last 10 years on the experience and motivations of volunteers. This research has concerned initiatives such as Sms-lifesavers and community first responders (CFR) [3–8]. This research has typically been conducted on small samples of volunteers using qualitative approaches such as interviews or focus groups [9–12]. As a result, many themes have been proposed as important for how volunteers experience their role and their motivation for continuing. However, the relative importance of the different themes and whether there are common factors underlying some of them has not been studied directly. Further, one area which has not been explored to date is how volunteers experience the alerts they receive and being dispatched to emergencies, and how this in turn impacts their motivation. Högstedt and colleagues explicitly identify a need for further research on how the frequency of alerts impact the motivation to continue as volunteers [13].

Högstedt and colleagues studied Sms-lifesavers motivation through categories derived from Self-determination theory (SDT) and found that Sms-lifesavers were mainly motivated by intrinsic factors [13]. The basic assumption for SDT is that humans are inclined to develop themselves and form a unified self, where different characteristics of the self as well as social relationships to other people are connected and integrated with each other [14, 15]. Bidee and colleagues further showed that for volunteers within healthcare, the volunteers’ feelings of inclusion within their volunteering group were related to intrinsic motivation, mediated by satisfaction of the needs of competence and relatedness [16].

In the current study, a survey was carried out to explore the experiences and motivation of volunteers related to retention, taking alerts into consideration. The survey had a broad and exploratory focus based on previous research about volunteers in first response, as well as the impact of alerts, specifically so-called alarm fatigue [17, 18]. The goals of the study were to (1) explore common factors underlying previously identified themes important for volunteer retention, (2) examine how such factors relate to volunteers’ motivation to continue volunteering, and (3) investigate how alerts and alarm fatigue relate to volunteers’ motivation to continue volunteering.

## Materials and methods

### Setting

Eligible participants were registered Sms-lifesavers or CIPs over the age of 18. Sms-lifesavers (Sw. “Sms-livräddare”) and civilian first responders (Sw. “civil insatsperson” or “CIP”) are both nation-wide, Swedish volunteer initiatives. In the Sms-lifesaver system, Swedish emergency dispatch centres can alert registered volunteers trained in cardiopulmonary resuscitation by a mobile-phone application in cases of suspected out-of-hospital cardiac arrest (OHCA) to aid prior to emergency medical services (EMS) arrival [3]. In the CIP system, trained volunteers who work together with municipal emergency services are alerted through mobile phones to provide support prior to emergency services arrival [4]. CIPs are dispatched to medical emergencies, such as OHCAs and drownings, as well as certain traffic accidents and fires. Participants were recruited to respond to an open, online survey, hosted at Linköping University, via emails sent to a mailing list for Sms-lifesavers and through Facebook-groups for CIPs.

### Participants

A total of 5347 volunteers responded to the survey, of which 5001 were Sms-lifesavers, 69 CIPs, and 277 both. There are currently over 100,000 registered Sms-lifesavers in Sweden. The number of CIPs are likely less than 1000, although no official records are kept on the number of CIP volunteers. The study followed the principles of the Declaration of Helsinki and was exempt from ethics approval in accordance with Swedish law. The participants received and digitally signed an informed consent form that contained information about the purpose of the study, estimated time to complete the survey, data storage procedures, and contact information to the responsible researchers for questions. The participants were also instructed to only answer the survey once, even if they had received multiple links (e.g., via the email list and via a Facebook group).

### Study design

The survey consisted of 48 items. Of these, 46 items were based on background literature; 32 based on themes brought up in prior research regarding volunteers’ experience and motivation as well as alarm fatigue research (see Additional file 1: Supplementary material 1), and 14 derived from SDT. For the SDT items, six items regarded basic needs satisfaction and were adapted from the “Basic Psychological Need Satisfaction and Frustration Scale—Work Domain” [19, 20], and eight items were adapted from the “Volunteer Motivation Scale” [21], with two items each for external, introjected and identified regulation and two for intrinsic motivation. In total, 30 of the aforementioned 46 items considered experience and motivation in general and 16 specifically in relation to alerts. Finally, one item considered motivation to continue volunteering, inspired by Wu and colleagues [22], and one considered intention to quit, adapted from Haivas and colleagues [23] and Millette and Gagné [21]. All items were graded on a 7-point Likert scale. At the end of the survey, participants had the opportunity to leave general comments. All items were written in Swedish.

Background information included age, gender, and whether the respondent had been active in a professional emergency service such as police, EMS, or rescue service. The survey asked whether the respondents live in a rural area (< 5000 inhabitants), smaller city (5000–50,000 inhabitants) or larger city (> 50,000 inhabitants), how many alerts they had received, accepted, and acted on in the last year, and for how long they had been volunteers. Respondents who were registered volunteers but had not received or acted on alerts yet were asked to answer with an estimation of how they believed they would react.

### Statistical analysis

The analysis had three stages: (1) Exploratory factor analysis (EFA) to identify latent factors, (2) multiple regression analysis using the identified factors to predict motivation to continue volunteering, and (3) between-group comparisons. The EFA factor extraction method used was maximum likelihood with oblimin rotation. Factors with an eigenvalue > 1 were included, and scree plots were inspected to make sure the cut-off point was reasonable. Bartlett scores was used to save factor scores, and missing values were replaced by means. Keiser–Meyer–Olkin (KMO) measure of sampling adequacy and Bartlett’s test of sphericity were used to assess whether the data was suitable for factor analysis. The regression analysis then used the saved factor scores to predict motivation to continue volunteering. Forced entry was used for the regression model. Finally, the between-group comparisons were based on four selected variables: respondents who had received alerts versus respondents who had not; respondents who had acted on alerts versus respondents who had not; respondents who had been active in a professional emergency service versus respondents who had not; and between respondents living in rural areas, smaller cities, and larger cities. Two-tailed t-tests were used for independent variables with two levels and ANOVA for variables with more than two levels. All analyses were performed using SPSS version 28.0.0.0.

## Results

Participants who reported that they did not fill out the survey properly or had already quit being an active volunteer (n = 23) and participants who did not fill out half or more of the 48 items (n = 155) were excluded from the data set, leaving 5169 participants for data analysis.

### Demographics

The participants had a mean age of 47.4 years (*SD* = 13.1). Of the participants, 2367 reported being women, 2789 men, four “other” and nine did not want to declare gender. Most participants (*n* = 2298) had been active in a professional emergency service, while 1816 had not. Concerning area of living, 1468 lived in a rural area, 1250 in a smaller city and 2433 in a larger city. Looking at alerts and acting on alerts, 1923 participants had not *received* an alert in the last year while 3246 had, and 3284 had not *acted* on alerts in the last year while 1885 had. On average, the volunteers reported receiving 2.36 alerts (*SD* = 6.67) and acting on 1.03 alerts (*SD* = 4.77) per year. The participants had on average been active volunteers for 3.48 years (*SD* = 3.31).

### Exploratory factor analysis

The KMO measure of sampling adequacy had a score of 0.89 and Bartlett’s test of sphericity was significant, χ^2^(1035) = 89,054.48, *p* < 0.001, suggesting that the data was suitable for factor analysis. The EFA resulted in 12 factors that accounted for 63.6% of the variance, see Table 1.

**Table 1:**
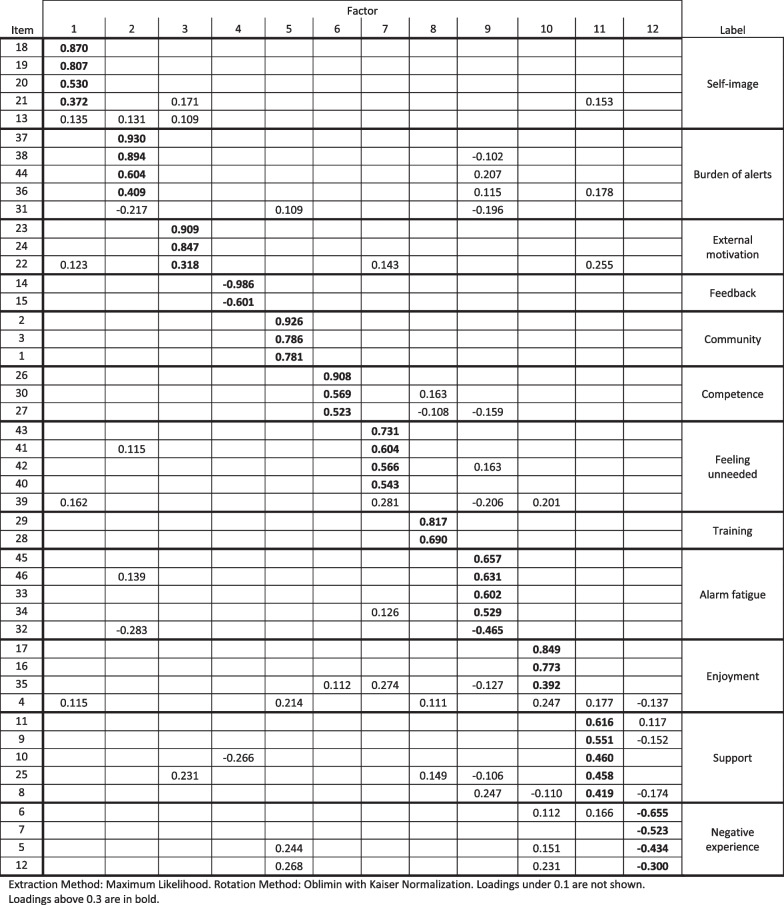
Factor loadings from the survey

The factors were named based on the theme that was deemed to be in common for all related items in the questionnaire. For example, items 2 (”I feel that I help people in my community in my role as volunteer”), 3 (“It is meaningful to help others by being a volunteer”) and 1 (“I feel that I help society by being a volunteer”) all loaded onto one factor, “Community”. Another example, items 26 (“I feel that I have the knowledge and skill necessary to help in some emergencies”), 30 (“I feel confident that I can manage everyday emergencies through the training I have received”), and 27 (“I feel unsure that I have the ability to help in some emergencies”, reverse coded) loaded onto “Competence”. Table 2 contains a description of each factor based on the items that constitute the factor. The original questionnaire items referred to in Table 1 are available in Additional file 1: Supplementary material 2.

**Table 2:** Description of the factors from the factor analysis

Factor	Description
Self-image	Feeling pride about volunteering and the volunteering initiatives they are part of. Perceiving it as personally important to volunteer, and that volunteering is a part of one’s self-image
Burden after alerts	Whether letting go after the alert in case of either a missed, turned down, or accepted and acted on alert is perceived as challenging. Also concerns guilt of not being able to act on alerts
External motivation	If conveying a positive image of oneself to others and receiving recognition from others is a motivating factor, and if receiving public recognition for their contributions would be motivating
Feedback	Wanting to know the outcome after acting on an alert, and whether knowing outcome would be motivating. Because the items load negatively, a high factor score indicates not wanting to know outcome and not being motivated by knowing the outcome
Community	Perceiving that they are helping their community by volunteering, and if helping others by volunteering feels meaningful
Competence	Whether one feel competent for the role as volunteer and in handling emergencies in everyday life. The factor also includes whether one feels doubt in being able to help in certain situations (this item was reverse coded)
Feeling unneeded	If not feeling needed at an emergency site would be frustrating and demotivating, and if they feel less inclined to act on alerts where they do not think they will be needed. Also, whether it would be demotivating to rarely receive alerts
Training	The perceived importance of receiving extensive initial training and continuous training for the role
Alarm fatigue	Whether alerts are perceived as burdening and stressing, and if receiving alerts at any time of the day would be strenuous. The factor also includes if receiving too many alerts would impact their willingness to volunteer, and if they experience a pressure to respond to alerts
Enjoyment	If being a volunteer and receiving alerts are fun, and whether being a volunteer is an enjoyment
Support	Wanting to have available support from psychologists and feedback from professionals, the need for support from family and friends, importance of meeting and talking to other volunteers about the role and whether the role can be psychologically or emotionally tough
Negative experience	Feeling that other people in the volunteering initiative care for the person, if the role as volunteer has fulfilled their expectation, and whether it has been a positive experience. The items were phrased in terms of positive experience but all load negatively, meaning that the factor measures negative experience rather than positive

### Motivation to continue

Overall, the descriptive statistics indicate high motivation to continue as a volunteer in the sample, with a mean score of 6.41 (SD = 1.049) on a scale from 1 (low motivation to continue) to 7 (high motivation to continue). The regression analysis predicting motivation to continue volunteering based on factor scores produced a model accounting for 40.2% of the variance in the data, *R*^2^ = 0.402, *F*(12,4991) = 279.87, *p* < 0.001. All individual predictors were significant (see Table 3). Positive beta-values indicate that higher scores on that factor is associated with increased motivation to continue as a volunteer, whereas negative values indicate that higher factor scores are associated with decreased motivation to continue. Of the predictors, “Community” had the strongest influence on the model (beta 0.31), followed by “Self-image” (0.17), “Alarm fatigue” (− 0.16), “Competence” (0.15), and “Negative experience” (− 0.10). The remaining factors had beta-weights of less than ± 0.10.

**Table 3:** Regression analysis

	β	*t*	*p*
Constant		557.90	< 0.001
Self-image	0.17	12.12	< 0.001
Burden after alerts	0.04	3.24	< 0.01
External motivation	− 0.05	− 4.14	< 0.001
Feedback	− 0.03	− 2.71	< 0.01
Community	0.31	24.19	< 0.001
Competence	0.15	12.11	< 0.001
Feeling unneeded	− 0.08	− 6.95	< 0.001
Training	0.06	5.12	< 0.001
Alarm fatigue	− 0.16	− 12.18	< 0.001
Enjoyment	0.08	5.55	< 0.001
Support	− 0.07	− 5.79	< 0.001
Negative experience	− 0.10	− 8.62	< 0.001

### Comparison between groups

The statistical test results of between-group differences are reported in Table 4. Volunteers who had *received* alerts had higher scores on “Feeling unneeded” compared to those who had not received an alert. Volunteers who had *acted* on alerts had higher scores on “Competence”, and “Feeling unneeded”, and lower scores on “Burden after alerts” and “Negative experience” as compared to those who had not acted on an alert. Volunteers who had been active in professional emergency services scored higher on the “Competence” factor, and lower on the “Burden after alerts”, “Alarm fatigue” and “Support” factors. For area of living, post hoc tests with Tukey corrections showed that volunteers in rural areas and smaller cities did not differ significantly but both rural areas and smaller cities had higher factors scores on “Community” as compared to larger cities, *F*(2, 5166) = 13.858, *p* < 0.001, *η*^2^ = 0.005. Volunteers in larger cities had higher scores on “Feeling unneeded” as compared to rural areas and smaller cities, *F*(2, 5166) = 15.350, *p* < 0.001,* η*^2^ = 0.006.

**Table 4:** Group comparisons

Dependent variable	*M *(*SD*)		*t*	*df*	*p*	Cohen’s *d*
	Has received alert	Has not received alert				
Burden after alerts	− 0.018 (1.076)	0.030 (1.006)	1.57	5167	0.115	0.05
Feeling unneeded	0.047 (1.148)	− 0.079 (1.134)	− 3.83	5167	< 0.001**	− 0.11
Alarm fatigue	0.020 (1.110)	− 0.034 (1.125)	− 1.67	5167	0.096	− 0.05
	Has acted on alert	Has not acted on alert				
Burden after alerts	− 0.069 (1.039)	0.040 (1.055)	3.57	5167	< 0.001**	0.10
Competence	0.169 (0.990)	− 0.097 (1.149)	− 8.42	5167	< 0.001**	− 0.24
Feeling unneeded	0.124 (1.176)	− 0.071 (1.119)	− 5.91	5167	< 0.001**	− 0.17
Support	0.007 (1.200)	− 0.004 (1.159)	− 0.34	5167	0.736	− 0.01
Negative experience	− 0.389 (1.096)	0.223 (1.216)	18.05	5167	< 0.001**	0.52
	Professional background	Not professional background				
Burden after alerts	− 0.123 (1.032)	0.173 (1.045)	9.08	4112	< 0.001**	0.29
Competence	0.413 (0.872)	− 0.495 (1.122)	− 29.22	4112	< 0.001**	− 0.92
Alarm fatigue	− 0.070 (1.079)	0.116 (1.160)	5.30	4112	< 0.001**	0.17
Support	− 0.174 (1.203)	0.215 (1.086)	10.76	4112	< 0.001**	0.34

** Significance at* p* < 0.01

## Discussion

The factors obtained from factor analysis are overall in line with the themes identified in the prior research described in Additional file 1: Supplementary material 1. All items regarding alerts and dispatch except one formed three factors, “Burden after alerts”, “Feeling unneeded”, and “Alarm fatigue”. The only alert-related item that did not load on the alert factors concerned enjoyment of alerts and therefore loaded on the “Enjoyment” factor. These factors suggests that the relation between alerts and motivation to continue volunteering must consider more than only the frequency of alerts. “Alarm fatigue” includes not only the frequency of alerts, but also the timing (e.g., alerts during the night) and the intrusiveness in terms of the alerts interrupting the daily life of the volunteer. The “Burden after alerts” on the other hand concerns the mental effort required to not dwell on an alert that the volunteer did not act on, and potential associated feelings of guilt, regardless of the frequency.

The regression analysis showed that “Community”, “Self-image” and “Competence” were the strongest positive predictors for the motivation to continue. “Community” and “Self-image” relate to the feeling of doing something good, and that it feels important for the individual to personally to help others. This is in line with previous research on first-response volunteers, where helping the community and inherent satisfaction have been brought up as important motivators [10, 11, 24, 26]. The fact that “external motivation” had little impact on the motivation to continue indicates that individual’s intrinsic experience of helping is more important than external reinforcement. “Competence” being a strong predictor also indicates that feeling competent is important for motivation to continue. Consequently, providing opportunities for developing skill and competence may increase volunteer retention [6].

In terms of factors that decrease motivation to continue volunteering, the factor “Alarm fatigue” was the strongest negative predictor. “Alarm fatigue” contains items about receiving many alerts and receiving alerts at any time, which suggests that receiving too many alerts or alerts at inconvenient times have a negative impact on the motivation to continue. “Alarm fatigue” had a stronger impact compared to other alert-related factors such as “feeling unneeded” and “burden after alerts”. This might indicate that a more important factor for demotivation is whether the alerts in themselves are perceived as tiring, rather than feelings after receiving or acting on alerts. Improved dispatch algorithms that take alarm fatigue effects into account may improve volunteer retention, as might increased user control over when and how the volunteers receive alerts. “Negative experience” was also a strong negative predictor. This factor captures overall feelings of expectations not being met and that others in the volunteer initiative do not care about them. Providing accurate information on what a volunteer should expect from a specific initiative in terms of the role and the social structure of the initiative may therefore also improve retention.

Most between-groups comparisons showed a significant effect. This is to be expected due to the large sample size, and emphasis should be put on the effect sizes to interpret the findings. One comparison that stand out in this regard is the difference in perceived competence between volunteers who have been active in professional emergency services and those who have not. The difference is unsurprising, considering that professionals in emergency services receive substantial training and practical experience through their job and prior education. However, since perceived competence was one of the stronger predictors for motivation to continue volunteering, offering additional or re-occurring training for non-professional volunteers might have a positive effect on volunteer retention.

A second comparison that stands out is the difference in negative experience between volunteers who had acted on alerts in the last year versus volunteers who had not, where volunteers who had acted on alerts had a less negative experience. Further exploration of the data showed a difference with a similar effect size between volunteers who had received an alert in the last year versus volunteers who had not, where volunteers who had not received alerts had a more negative experience. One interpretation of these results is that not receiving any alerts whatsoever might lead to higher scores on the “Negative experience” factor. A possible implication of this is that some sort of encouragement or interaction with volunteers who do not receive any alerts might be helpful to improve their experience.

## Limitations

The factor analysis results are based on correlations, and it is therefore important to be cautious in drawing conclusions about causality based on these results. Furthermore, the study was exploratory and therefore included many factors at a general level. It would be advisable to perform further studies based on the current findings to examine the causal relationships and factors in more detail.

The sample for the study consisted of active volunteers, which might lead to a selection bias. For volunteers who have already quit, different factors might be connected to them no longer volunteering. However, prior studies on volunteer attrition indicates that the results of the current study are likely to generalize to volunteers who have quit [24]. Rørtveit and Meland performed a longitudinal study on different groups of CFRs and noted that volunteers in groups with high withdrawal considered the task to be burdensome [24]. This is in line with the strong, negative impact of the “Alarm fatigue” factor in the current study.

## Conclusions

To conclude, 12 factors related to volunteers’ experience of and motivation for volunteering in first response were identified, and their relation to motivation for continued volunteering as well as between-group differences on the factors were analysed. Based on the results, the following could be considered by organizations involved with volunteer first responders:

- Alarm fatigue is a concern for maintaining volunteers. Efforts should be made to develop dispatch algorithms that do not alert more volunteers than is needed, and to offer the volunteers the option to set “away” periods when they do not wish to receive alerts.
- Support after being dispatched is important, especially for non-professional volunteers. Routines should be developed so that dispatched volunteers can ask to know outcomes (after necessary release approvals are secured). On-demand or screening counselling, or some form of after-action review opportunities could also be considered.
- Training and refresher training offered to non-professional volunteers should also aim to support the volunteer’s confidence in their competency.

### Supplementary Information

**Additional file 1:** Supplementary material 1 and 2.

## Data Availability

The datasets used in the current study are available from the corresponding author on reasonable request.

## References

[1] Mojir KY, Pilemalm S. A framework for “new actors” in emergency response systems. In: Comes T, Fiedrich F, Fortier S, Geldermann J, Müller T, editors. ISCRAM 2013 conference proceedings—10th international conference on information systems for crisis response and management. Baden-Baden: Karlsruher Institut fur Technologie; 2013, p. 741–6.

[2] Matinrad N, Reuter-Oppermann M (2022). A review of initiatives for the management of daily medical emergencies prior to the arrival of emergency medical services. CEJOR.

[3] Ringh M, Rosenqvist M, Hollenberg J (2015). Mobile-phone dispatch of laypersons for CPR in out-of-hospital cardiac arrest. N Engl J Med.

[4] Pilemalm S (2022). Hur expanderar vi konceptet civila insatspersoner: Att hantera organisatoriska och IT-relaterade hinder.

[5] Berglund E, Olsson E, Jonsson M (2022). Wellbeing, emotional response and stress among lay responders dispatched to suspected out-of-hospital cardiac arrests. Resuscitation.

[6] Phung V-H, Trueman I, Togher F, Orner R, Siriwardena AN (2017). Community first responders and responder schemes in the United Kingdom: systematic scoping review. Scand J Trauma Resusc Emerg Med.

[7] Ries ES, Kragh AR, Dammeyer J, Folke F, Andelius L, Hansen CM (2021). Association of psychological distress, contextual factors, and individual differences among citizen responders. J Am Heart Assoc.

[8] Zijlstra JA, Beesems SG, De Haan RJ, Koster RW (2015). Psychological impact on dispatched local lay rescuers performing bystander cardiopulmonary resuscitation. Resuscitation.

[9] Barry T, Guerin S, Headon M, Bury G (2020). GPs who volunteer to be first responders for out-of-hospital cardiac arrest: a qualitative study. Eur J Gen Pract.

[10] Davies E, Maybury B, Colquhoun M, Whitfield R, Rossetti T, Vetter N (2008). Public access defibrillation: psychological consequences in responders. Resuscitation.

[11] Phung V-H, Trueman I, Togher F, Ørner R, Siriwardena AN (2018). Perceptions and experiences of community first responders on their role and relationships: qualitative interview study. Scand J Trauma Resusc Emerg Med.

[12] Timmons S, Vernon-Evans A (2013). Why do people volunteer for community first responder groups?. Emerg Med J.

[13] Högstedt A, Thuccani M, Carlström E (2022). Characteristics and motivational factors for joining a lay responder system dispatch to out-of-hospital cardiac arrests. Scand J Trauma Resusc Emerg Med.

[14] Ryan RM, Deci EL (2000). Self-determination theory and the facilitation of intrinsic motivation, social development, and well-being. Am Psychol.

[15] Ryan RM, Deci EL, Deci EL, Ryan RM (2002). Overview of self-determination theory: an organismic-dialectical perspective. Handbook of self-determination research.

[16] Bidee J, Vantilborgh T, Pepermans R, Willems J, Jegers M, Hofmans J (2017). Daily motivation of volunteers in healthcare organizations: relating team inclusion and intrinsic motivation using self-determination theory. Eur J Work Organ Psychol.

[17] Cvach M (2012). Monitor alarm fatigue: an integrative review. Biomed Instrum Technol.

[18] Lewandowska K, Weisbrot M, Cieloszyk A, Mędrzycka-Dąbrowska W, Krupa S, Ozga D (2020). Impact of alarm fatigue on the work of nurses in an intensive care environment—a systematic review. Int J Environ Res Public Health.

[19] Chen B, Vansteenkiste M, Beyers W (2015). Basic psychological need satisfaction, need frustration, and need strength across four cultures. Motiv Emot.

[20] Schultz PP, Ryan RM, Niemiec CP, Legate N, Williams GC (2015). Mindfulness, work climate, and psychological need satisfaction in employee well-being. Mindfulness.

[21] Millette V, Gagné M (2008). Designing volunteers’ tasks to maximize motivation, satisfaction and performance: the impact of job characteristics on volunteer engagement. Motiv Emot.

[22] Wu Y, Li C, Khoo S (2016). Predicting future volunteering intentions through a self-determination theory perspective. VOLUNTAS Int J Volunt Nonprofit Organ.

[23] Haivas S, Hofmans J, Pepermans R (2013). Volunteer engagement and intention to quit from a self-determination theory perspective. J Appl Soc Psychol.

[24] Rørtveit S, Meland E (2010). First responder resuscitation teams in a rural Norwegian community: sustainability and self-reports of meaningfulness, stress and mastering. Scand J Trauma Resusc Emerg Med.

[25] Barry T, Guerin S, Bury G (2019). Motivation, challenges and realities of volunteer community cardiac arrest response: a qualitative study of ‘lay’ community first responders. BMJ Open.

[26] Heffernan E, Mc Sharry J, Murphy A (2021). Community first response and out-of-hospital cardiac arrest: a qualitative study of the views and experiences of international experts. BMJ Open.

[27] Ramsell E, Pilemalm S, Granberg TA. Using volunteers for emergency response in rural areas—network collaboration factors and IT support in the case of enhanced neighbors. In: Comes T, Bénaben F, Hanachi C, Lauras M, Montarnal A, editors. Proceedings of the 14th international conference on information systems for crisis response and management. Albi: ISCRAM Association; 2017, p. 985–95.

[28] Roberts A, Nimegeer A, Farmer J, Heaney DJ (2014). The experience of community first responders in co-producing rural health care: in the liminal gap between citizen and professional. BMC Health Serv Res.

[29] Axelsson Å, Herlitz J, Ekström L, Holmberg S (1996). Bystander-initiated cardiopulmonary resuscitation out-of-hospital. A first description of the bystanders and their experiences. Resuscitation.

[30] Chen H-H, Chiang W-C, Hsieh M-J (2020). Experiences and psychological influences in lay rescuers performing bystander cardiopulmonary resuscitation: a qualitative study. J Acute Med.

[31] Harrison-Paul R, Timmons S, van Schalkwyk WD (2006). Training lay-people to use automatic external defibrillators: are all of their needs being met?. Resuscitation.

[32] Haskins B, Nehme Z, Dicker B (2021). A binational survey of smartphone activated volunteer responders for out-of-hospital cardiac arrest: availability, interventions, and post-traumatic stress. Resuscitation.

[33] Kragh AR, Andelius L, Gregers MT (2021). Immediate psychological impact on citizen responders dispatched through a mobile application to out-of-hospital cardiac arrests. Resusc Plus.

[34] Mathiesen WT, Bjørshol CA, Braut GS, Søreide E (2016). Reactions and coping strategies in lay rescuers who have provided CPR to out-of-hospital cardiac arrest victims: a qualitative study. BMJ Open.

[35] Axelsson Å, Herlitz J, Karlsson T (1998). Factors surrounding cardiopulmonary resuscitation influencing bystanders’ psychological reactions. Resuscitation.

[36] Schmid F, Goepfert MS, Reuter DA (2013). Patient monitoring alarms in the ICU and in the operating room. Crit Care.

[37] Sowan AK, Tarriela AF, Gomez TM, Reed CC, Rapp KM (2015). Nurses’ perceptions and practices toward clinical alarms in a transplant cardiac intensive care unit: exploring key issues leading to alarm fatigue. JMIR Hum Factors.

[38] Gazzale L (2019). Motivational implications leading to the continued commitment of volunteer firefighters. Int J Emerg Serv.

[39] Lantz E, Runefors M (2021). Recruitment, retention and resignation among non-career firefighters. Int J Emerg Serv.

[40] Compion S, Meijs L, Cnaan RA, Krasnopolskaya I, von Schnurbein G, Abu-Rumman S (2022). Repeat and non-returning volunteers: the promise of episodic events for volunteer recruitment and retention. VOLUNTAS Int J Volunt Nonprofit Organ.

[41] Hallmann K, Harms G (2012). Determinants of volunteer motivation and their impact on future voluntary engagement: a comparison of volunteer’s motivation at sport events in equestrian and handball. Int J Event Festiv Manag.

[42] Malinen S, Algera P, Mankkinen T (2020). Volunteer motivations in the Finnish fire service. Int J Emerg Serv.

[43] Pozzi M, Meneghini AM, Marta E (2019). Does volunteering at events motivate repeat engagement in voluntary service? The case of young adult volunteers at EXPO Milan 2015. Test Psychom Methodol Appl Psychol.

[44] Hyde MK, Dunn J, Bax C, Chambers SK (2016). Episodic volunteering and retention: an integrated theoretical approach. Nonprofit Volunt Sect Q.

