# Supplementary material for: What motivates and demotivates emergency response volunteers? A survey-based factor analysis study
Source: Scand J Trauma Resusc Emerg Med. 2023 Aug 12;31:38. doi: 10.1186/s13049-023-01101-0 (PMC10422740; doi:10.1186/s13049-023-01101-0)
Supplement: Supplementary file 1 — Additional file 1: Supplementary material 1 and 2. [file 13049_2023_1101_MOESM1_ESM.docx]

**Supplementary material 1**

Themes extracted from prior research regarding volunteers’ experience and motivation as well as alarm fatigue research.

| **Theme** | **Description** |
| --- | --- |
| **Experience and motivation of volunteers** | |
| Community | One often identified motivation for volunteering is helping others in the community and giving back to the community.^6,9,10,12,25-28^ |
| Inherent motivation | The possibility of saving lives and make a difference for people through volunteering can be satisfying and thereby inherently motivating. Altruistic and empathic personality traits are connected to volunteering.^10,11,24,26^ |
| External motivation | Some professionals within healthcare feel obliged to help due to having the knowledge and skills needed. Being provided with emergency training have been seen as a positive, as well as experience of emergency work valuable for future employment.^9,28^ |
| Psychological impact | The situations volunteers respond to can be emotionally and psychologically challenging, which may cause psychological and emotional distress leading to negative psychological reactions such as flashbacks. Some short-term psychological impact, stress and emotional discomfort have been reported by volunteers after acting on alarms, although often at low levels. Long-term psychological impact such as persisting PTSD symptoms are rarely experienced.^1,5,7-9,24,25,28,30-34^ |
| Positive experience | Performing CPR are generally rated as a positive experience, and most report being willing to perform CPR again. Volunteers also reported a willingness to continue after being dispatched to incidents. Successful outcomes have been reported to bring satisfaction and intrinsic pleasure, as well as overall satisfaction, self-esteem, and pride from helping others.^5,7,8,10,11,26,30,32,33,35^ |
| Need for support | Most volunteers report talking to someone, such as family or friends, about their experience performing CPR. Some volunteers reported seeking professional support, either with regards to mental health or feedback on their performance. Volunteers generally appreciated debriefing after resuscitation attempts, as well as having somebody available to talk to if needed. Both formal forms of support and informal, such as debriefing with other volunteers, have been appreciated by volunteers.^8,10,26,29,30,34,35^ |
| Systems for providing support | The need for systems to provide support for volunteers have been proposed. This support is suggested to be able deal with both psychological aspects and questions about performance at the emergency. Informal support systems that for example provide opportunities to speak to peers have also been emphasised as important.^8,11,26,27,29,30-35^ |
| Training | Volunteers have reported both wanting more training and practical training, as well as repeated and continuous training to keep their competence up to date. Volunteers who are not healthcare professionals reported being less prepared for emergency situations.^6,25,27,29,30,32^ |
| Knowing outcome | Volunteers have reported an interest in learning outcomes of victims in emergencies. Getting to know the outcome in situations with a negative outcome might lead to negative reactions due basing evaluation of performance to the outcome. However, not knowing the outcome might also have negative consequences.^26,28,30,31,34,35^ |
| Social aspects | For CFRs, social aspects of volunteering such as getting to know other volunteers and being involved in the community have been reported as motivating and supporting. However, social aspects can also be challenging, e.g. by conflicts in volunteer groups.^25,26,28^ |
| Burden of on-call | Constantly being on call and being able to be called out at any time have been reported as stressful and burdensome. Not being able to respond to calls might cause guilt. Being able to mentally “switch off” between calls has been reported as important. Night-time callouts were especially stressful, possibly causing alarm fatigue.^4,10,25,28^ |
| Number of alerts | Receiving very few alerts has been reported as demotivating.^12^ |
| Returning to normal | Resetting and going back to normal after acting on alerts can be challenging, due to stress from the situation. Restlessness, sleep deprivation, weight loss and other psychological impacts have also been reported after acting on alerts.^5,9,30,34^ |
| Frustration of acting on alerts needlessly | Acting on alerts without getting to help due to EMS arriving prior to the volunteer have been reported as frustrating.^11^ |
| **Alarm fatigue** | |
| Alarm fatigue | Excessive alarms false alarms, burdensome alarms and non-actionable alarms can cause reduced responding to alerts or turning alarm systems off completely. Alerts might also cause stress by disrupting ongoing activities.^17,18,36,37^ |
| **Retention of volunteers** | |
| Community | Having close connections to the community and both being committed to and considering it important to help the community is connected to retention of volunteer firefighters.^38,39^ |
| Motivation | Motivation has been identified as important for retention. Social and utilitarian motives for volunteering were associated with continued volunteering, as well as altruistic motives such as caring for others and personal growth.^40-43^ |
| Satisfaction | High satisfaction with the volunteering experience, both regarding overall satisfaction, contentment with the organiser and comfort with tasks, is related to higher retention. This goes for both novice and more experienced volunteers. Helping victims of different incidents and helping new members of the volunteering team both were rewarding and satisfying for volunteers.^38,39,40,44^ |
| Comradery | Social factors have been pointed out as important especially for new volunteers. Commitment to the organisation is also important for retention, especially for long term volunteers. For volunteer firefighters, comradery with the other volunteers supports retention. Disputes within the volunteer group and feelings of exclusion may cause volunteers to quit.^38,39,44^ |
| Support from supervisor | Getting support and feedback from the organisation and supervisors affect both retention and overall satisfaction. Having a just organisation and a positive work climate are also considered important.^39^ |

**Supplementary material 2**

Items in the survey, in Swedish with English translation. Items marked with an asterisk were reverse coded prior to analysis.

| 1 | Rollen som frivillig kan vid vissa tillfällen vara psykologiskt eller emotionellt jobbig. | Being a volunteer can occasionally be psychologically or emotionally draining. |
| --- | --- | --- |
| 2 | Jag känner att det är viktigt att få stöd i min roll som frivillig av familj och vänner. | I feel that is important to receive support in my role as volunteer from family and friends |
| 3 | Jag vill ha möjlighet att få återkoppling från professionella inom området på mina insatser vid utryckningar. | I want the option to receive feedback from professionals in the area concerning my actions during emergency responses. |
| 4 | Jag vill ha möjlighet att få stöd i min roll som frivillig av professionella psykologer. | I want the option to receive support in my role as volunteer from professional psychologists. |
| 5 | Det har överlag varit en positiv upplevelse att vara frivillig. | It has overall been a positive experience to be a volunteer. |
| 6 | Jag känner en skyldighet till att ställa upp som frivillig. | I feel a duty to be a volunteer. |
| 7 | Jag skulle vilja veta hur det gick för de drabbade i räddningsinsatser jag deltagit i. | I would like to know what happened to those affected in the emergency responses I have participated in. |
| 8 | Att få veta hur det gick för de drabbade skulle vara motiverande för att fortsätta vara frivillig. | To know what happened to those affected would be motivating to continue as a volunteer. |
| 9 | Jag upplever det som roligt att vara frivillig. | I find volunteering fun. |
| 10 | Jag upplever att det är ett nöje för mig att vara frivillig. | I find it enjoyable to be a volunteer. |
| 11 | Att vara frivillig får mig att känna mig stolt och värdefull som person. | To be a volunteer makes me feel proud and valuable as a person. |
| 12 | Jag känner mig stolt över att vara en del av min/mina frivilligorganisation(er). | I feel proud to be part of my volunteer organization(s) |
| 13 | Jag känner att det är viktigt för mig personligen att vara frivillig och hjälpa till vid nödsituationer. | I feel that it is important for me personally to be a volunteer and help in emergencies. |
| 14 | Jag är frivillig eftersom det har blivit en viktig del i min bild av mig själv. | I am a volunteer because it has become an important part of my own image of myself. |
| 15 | Det skulle vara motiverande att få offentligt erkännande för det vi frivilliga bidrar med till samhället. | It would be motivating to receive public recognition for what we volunteers contribute to society. |
| 16 | Jag är frivillig för att andra ska få en positiv bild av mig som person. | I am a volunteer so that others will have a positive image of me as a person. |
| 17 | Jag är frivillig för att få erkännande från andra. | I am a volunteer to receive recognition from others. |
| 18 | Jag tycker att det är viktigt att få träffa andra frivilliga och kunna prata om mina upplevelser med dem. | I feel that it is important to meet other volunteers and talk about my experiences with them- |
| 19 | Jag känner att jag har den kunskap och de färdigheter jag behöver för min roll. | I feel that I have the knowledge and skills I need to perform my role. |
| 20 | *Jag känner mig tveksam till om jag har förmågan att hjälpa till i vissa utryckningssituationer. | *I am doubtful that I have the capability to help in some emergency situations. |
| 21 | Jag känner att det är viktigt att ha fått omfattande träning innan man börjar som frivillig. | I feel that it is important to receive rigorous training before becoming a volunteer. |
| 22 | Jag känner att det är viktigt att kontinuerligt få träning för att upprätthålla de förmågor jag lärt mig genom tidigare träning. | I feel that it is important to continuously receive training to maintain the skills I have previously learnt. |
| 23 | Jag känner mig trygg med att jag till vardags kan hantera vissa nödsituationer genom den träning jag fått. | I feel confident that I in everyday situations can handle emergencies thanks to the training I have received. |
| 24 | Jag känner mig fri att välja om jag vill svara på larm eller inte. | I feel free to choose if I should respond to an alarm or not. |
| 25 | *Jag upplever en press att svara på larm. | *I feel a pressure to respond to alarms. |
| 26 | Det skulle kännas jobbigt att kunna få larm vid vilken tidpunkt som helst. | It would feel challenging to receive alarms at any time of the day. |
| 27 | Att få för mycket larm skulle påverka min vilja att vara frivillig. | To receive too many alarms would affect my commitment to being a volunteer. |
| 28 | Det känns roligt att få larm. | It is fun to receive alarms. |
| 29 | Efter en genomförd insats känner jag att jag har svårt att sluta tänka på insatsen efteråt. | After a completed emergency response I find it difficult to stop thinking about the emergency. |
| 30 | Efter att ha tackat nej till ett larm känner jag att jag har svårt att sluta tänka på larmet efteråt. | After having declined an alarm I find it difficult to stop thinking about the alarm. |
| 31 | Efter att ha missat ett larm så har jag svårt att sluta tänka på larmet efteråt. | After having missed an alarm I find it difficult to stop thinking about the alarm. |
| 32 | Det känns motiverande att få larm. | It feels motivating to receive alarms. |
| 33 | Jag skulle vara mindre motiverad att vara frivillig om jag väldigt sällan fick larm. | I would be less motivated to be a volunteer if I rarely received any alarms. |
| 34 | Jag skulle uppleva det som frustrerande att rycka ut på ett larm utan att få hjälpa till. | I would find it frustrating to respond to an alarm without being allowed to help. |
| 35 | Jag känner mig i vissa fall mindre benägen att rycka ut på larm för att jag tror att jag inte kommer behövas på det larmet. | I feel, in some situations, less inclined to respond to alarms because I believe I will not be needed for that emergency. |
| 36 | Att känna att jag inte behövs på utryckningar skulle göra mig mindre motiverad till att vara frivillig. | To feel that I am not needed during an emergency would make me less motivated to continue as a volunteer. |
| 37 | Jag känner skuld när jag inte kan rycka ut på larm. | I feel guilt when I cannot respond to alarms. |
| 38 | Jag upplever det som betungande att få larm. | I find that alarms are a burden. |
| 39 | Jag upplever det som stressande att få larm. | I find alarms stressful. |
| 40 | Jag känner mig motiverad till att fortsätta vara frivillig. | I feel motivated to continue as a volunteer. |
| 41 | Jag tror jag kommer sluta vara frivillig inom det närmsta året. | I believe I will stop being a volunteer within the next year. |
